# Generalized Joint Hypermobility and Anxiety in Adolescents and Young Adults, the Impact on Physical and Psychosocial Functioning

**DOI:** 10.3390/healthcare9050525

**Published:** 2021-04-29

**Authors:** Janneke de Vries, Jeanine Verbunt, Janine Stubbe, Bart Visser, Stephan Ramaekers, Patrick Calders, Raoul Engelbert

**Affiliations:** 1 School of Physiotherapie, Amsterdam University of Applied Sciences, 1105 BD Amsterdam, The Netherlands; s.p.j.ramaekers@hva.nl; 2 Amsterdam UMC, Department of Rehabilitation, Amsterdam Movement Sciences, University of Amsterdam, 1105 AZ Amsterdam, The Netherlands; 3 Centre of Expertise Urban Vitality, Faculty of Health, Amsterdam University of Applied Sciences, 1105 BD Amsterdam, The Netherlands; b.visser2@hva.nl; 4 Department of Rehabilitation Medicine, CAPHRI, Functioning and Rehabilitation, Maastricht University Medical Center, 6229 HX Maastricht, The Netherlands; jeanine.verbunt@maastrichtuniversity.nl; 5 Adelante Center of Expertise in Rehabilitationand Audiology, 6432 CC Hoensbroek, The Netherlands; 6 Codarts, University of the Arts, 3012 CC Rotterdam, The Netherlands; jhstubbe@codarts.nl; 7 PErforming Artist and Athlete Research Lab (PEARL), 3012 CC Rotterdam, The Netherlands; 8 Department of General Practice, Erasmus MC University Medical Center, 3015 GD Rotterdam, The Netherlands; 9 Rotterdam Arts and Science Lab (RASL), 3012 CC Rotterdam, The Netherlands; 10 Department of Rehabilitation Sciences, Faculty of Medicine and Health Sciences, Ghent University, 9000 Ghent, Belgium; Patrick.Calders@ugent.be; 11 Amsterdam UMC, Department of Pediatrics, Emma Children’s Hospital, University of Amsterdam, Meibergdreef 9, 1105 AZ Amsterdam, The Netherlands

**Keywords:** hypermobility, adolescent, anxiety

## Abstract

The purpose of this study was to study the association between the presence of generalized joint hypermobility (GJH) and anxiety within a non-clinical high performing group of adolescents and young adults. Second, to study the impact of GJH and/or anxiety on physical and psychosocial functioning, 168 adolescents and young adults (mean (SD) age 20 (2.9)) were screened. Joint (hyper)mobility, anxiety, and physical and psychosocial functioning were measured. In 48.8% of all high performing adolescents and young adults, GJH was present, whereas 60% had symptoms of anxiety. Linear models controlled for confounders showed that adolescents and young adults with GJH and anxiety had decreased workload (ß (95%CI) −0.43 (−0.8 to −0.08), *p*-value 0.02), increased fatigue (ß (95%CI) 12.97 (6.3–19.5), *p*-value < 0.01), and a higher level of pain catastrophizing (ß (95%CI) 4.5 (0.5–8.6), *p*-value 0.03). Adolescents and young adults with only anxiety had increased fatigue (ß (95%CI) 11 (4.9–19.5). In adolescents and young adults with GJH alone, no impact on physical and psychosocial functioning was found. Adolescents and young adults with the combination of GJH and anxiety were significantly more impaired, showing decreased physical and psychosocial functioning with decreased workload, increased fatigue, and pain catastrophizing. Presence of GJH alone had no negative impact on physical and psychosocial functioning. This study confirms the association between GJH and anxiety, but especially emphasizes the disabling role of anxiety. Screening for anxiety is relevant in adolescents and young adults with GJH and might influence tailored interventions.

## 1. Introduction

Generalized joint hypermobility (GJH) is characterized by the ability to move beyond the “normal” limits of range of motion in multiple joints. GJH is not necessarily synonymous with complaints and could also be helpful to excel, for example, in sports like rhythmic gymnastics or dance, where GJH is highly prevalent [1,2]. The pressure to perform in elite sports can be overwhelming with a possible increase in risk of injury and psychological overload [3]. When GJH, as a non-symptomatic condition, is accompanied by chronic musculoskeletal pain and soft tissue injury, GJH is referred to as Joint Hypermobility Syndrome (JHS) [4]. JHS shows similarities with the hypermobile type of Ehlers–Danlos syndrome (EDS-HT) [5]. EDS-HT is described based on major criteria as GJH and skin involvement and minor criteria as chronic pain, recurrent dislocations, and a positive family history [6]. A revision of the clinical criteria of EDS in 2017 clarifies the diversity within hypermobility [7]. In EDS-HT, soft tissue frailty was added and is now referred to as hypermobile EDS (hEDS). The term Hypermobility Spectrum Disorder (G-HSD) is currently used for JHS and former EDS-HT patients who do not fulfill the new criteria (hEDS) [7].

There have been minimal studies pertaining to disease progression in GJH, h-EDS, and HSD, with the available studies primarily addressing physical functioning [8,9]. In the disease progression of hEDS/HSD from childhood, adolescence, and adulthood roughly three phases are proposed based on cross-sectional observations by Castori et al. [10]. The first phase, in childhood and adolescence, is dominated by an excessive amplification of tissue laxity and increased joint flexibility, while in adulthood phases, two and three describe next to physical factors like GJH, and psychosocial complaints like pain avoidance, anxiety, and depression. Phases two and three seem to have an increasing impact on quality of life accompanied by physical deconditioning and limited joint mobility [10]. However, this theory has not been verified yet. Current models in the GJH literature describe an association between GJH and anxiety but do not differentiate individuals with both GJH and anxiety from those with GJH alone [9,11]. The association between GJH and anxiety and the impact on physical functioning in healthy adolescents and young adults has not been studied in detail, whereas adolescents and young adults, in this specific lifespan, might be even more vulnerable for anxiety related disability, based on fear of negative evaluation or social phobias [12]. The impact might best be studied in a population in which hypermobility is highly prevalent with 57%, such as in young high performing dancers [1,2]. Hypermobility is common in high performance dancers because it is perceived as a sign of talent [13].

Our objective is to study the impact of GJH and anxiety on physical and psychosocial functioning within a non-clinical group of high performing adolescents and young adults. 

## 2. Materials and Methods

Two cohorts of first year students of a dance academy were screened on musculoskeletal complaints, and physical and psychosocial functioning a week before the start of their education in August 2015 and 2016. All students were enrolled after they successfully completed the regular selection and audition procedure. Students were eligible for inclusion when (1) no orthopedic, cardiopulmonary, rheumatological, or neurological conditions or disorders influencing physical performance were present, and (2) they were able to understand the questionnaires and to adhere to the protocol. 

From all participants, written informed consent was obtained. To create an open and safe environment, the screening took place outside the dance academy in the outpatient clinic of a university medical center without presence of staff of the dance academy. The outcomes were shared individually with the student without informing the dance academy, in order to secure the privacy. The study was approved by the Medical Ethical Committee of the Amsterdam University Medical Center (reference numbers W15_093#15.0110 and W16_237#16.277). 

Physiotherapy students performed the functional testing as assessors after having received intensive training. For three weeks, training of standardized operating procedures was performed by expert researchers with broad experience in screening GJH, and measurements were analyzed for inter-rater reliability. The expert researchers remained present during the measurements. In between, while resting, the questionnaires were filled in. 

### 2.1. Demographic and Body Characteristics

Data regarding age, gender, medication use, and injury history were obtained. Body characteristics such as standing height (cm), measured with a wall mounted stadiometer, and weight (kg), measured by a scale both measured without shoes and heavy clothing (rounded to the nearest centimeter and 0.1 kg) were collected, and BMI was calculated (kg/m^2^).

#### 2.1.1. Physical Functioning

Participants were asked if they suffered from a present injury or an injury in the past two weeks, which body part was affected, if it was traumatic, the duration, and if this was a recurrent injury. An injury was defined as “any physical complaint that enables you to participate in dance activities” [14].

Joint (hyper)mobility The Beighton score (BS) to measure the presence of local and generalized joint (hyper)mobility was performed as the first assessment of the measurement set not allowing the participants to have a warming-up phase. The BS consists of five clinical bilateral maneuvers of the little finger, thumb, elbow, and knee, each side scoring 1 point, and the spine also scoring 1 point, resulting in a total score of 9 points. Generalized joint hypermobility (GJH) was determined by a Beighton score ≥ 5, as described by Juul-Kristensen et al. The Beighton score is a validated instrument to measure hypermobility in adolescents and adults with an acceptable inter-rater reliability [15].

Muscle strength was measured by a hand-held dynamometer (Citec, Groningen, The Netherlands) according to a standardized protocol [16] and expressed in Newtons. Total muscle strength was presented as a composite score of strength tests in the ankle dorsal flexors, knee extensors, hip flexors, shoulder abductors, elbow flexors, and handgrip. The break method was conducted in the hip flexors, ankle dorsal flexors, elbow flexors, and the shoulder abductors. Regarding grip strength and muscle strength of the knee extensors, the make method was used [16,17]. Measurements were performed bilaterally three times, whereas the highest value of both sides was summed up and divided by two to create a mean muscle strength per muscle group. Values were compared to the reference’s values of adolescents [16]. The reliability of hand-held dynamometry in children, adolescents, and adults has been established with test–retest correlation coefficients ranging from 0.74 to 0.99.

Workload was measured by the steep ramp test (SRT) and performed at a protocolled electronically braked cycle ergometer (Lode Corival CPet; Lode B.V. Groningen, the Netherlands). After three-minutes of warming-up at 25 Watt (W), the ramp protocol [18] applied 20 W/10 s resistance, and the participant was instructed to maintain a pedaling frequency of 60 to 80 revolutions per minute (rpm). Peak performance was defined as the point at which despite strong verbal encouragement, the pedaling frequency dropped below 60 rpm. The maximum wattage was normalized for weight and expressed as W/kg. The SRT is a reliable and valid exercise test in adolescents and adults [18,19].

#### 2.1.2. Psychosocial Functioning and Pain Intensity

Anxiety and Depression were measured by the Hospital Anxiety and Depression Scale (HADS) [20]. The self-reported questionnaire consists of two subscales (anxiety and depression), each consisting of 7 items rated on a 4-point Likert scale from 0 to 3, with higher scores indicating higher levels of anxiety or depressive state. A subscale score equal or above 8 in the general population is an indicator of symptoms of anxiety or symptoms of depression [20]. Subscales showed good internal consistency (HADS anxiety Chronbach’s α = 0.83 (0.68–0.93); HADS depression (Chronbach’s α = 0.82 (0.67–0.90)) and good concurrent validity [20] in somatic (mostly cancer), psychiatric, primary care patients, and the general population, including both adults and adolescents.

Fatigue was quantified by the Checklist Individual Strength (CIS20) [21,22], a self-reported questionnaire consisting of 20 items rated on a 7-point Likert scale ranging from 1 to 7 with higher scores indicating higher levels of fatigue. Known cut-off for severe fatigue is a total score equal or above 40 [21]. The CIS20 has a good internal consistency of the total scale (Chronbach’s α = 0.90) and good validity in multiple groups [23]. 

Pain coping was defined by both pain catastrophizing, measured by the Pain Catastrophizing Scale (PCS) [24], and vigilance, measured by the Pain Vigilance and Awareness Questionnaire (PVAQ) [25]. The PCS asks the participants to reflect on past painful experiences and to indicate the degree to which they experienced each of 13 thoughts or feelings when experiencing pain. The self-reported questionnaire consists of 13 items rated on a 5-point Likert scale with higher scores indicating higher levels of catastrophizing. The PCS comprises subscales for rumination (4 items), magnification (3 items), and helplessness (6 items). The PVAQ measures the participant’s attention to pain itself and changes in pain. The self-reported questionnaire consists of 16 items, which are rated on a 6-point Likert scale with higher scores indicating higher levels of attention. Both the PCS and the PVAQ showed good internal consistency of the total scores (Chronbach’s α = 0.95 for PCS, Chronbach’s α = 0.94 for PVAQ) [24,25].

### 2.2. Data Analysis

Distribution of the data was checked, and if normality was confirmed by Kolmogorov–Smirnov mean, standard deviations and range were presented, whereas skewed data were presented as median (50th percentile) and interquartile range (25th and 75th percentile), and nominal data was presented as number and percentage. Data of the two cohorts (2015 and 2016) were combined, since an independent *t*-test, Mann Whitney-U, or the chi-square test for every variable showed no significant differences in demographic variables. Descriptive statistics stratified for gender were used to present the clinical characteristics and physical and psychosocial functioning. To study the impact of GJH and anxiety on physical functioning, either separately or in combination, four subgroups were constructed: (1) adolescents and young adults without GJH and anxiety (no anxiety/no GJH), (2) adolescents and young adults with GJH alone (GJH), (3) adolescents and young adults with anxiety alone (anxiety), and (4) adolescents and young adults with increased presence of both GJH and anxiety (GJH and anxiety). Univariate analysis was performed by comparing outcomes of physical and psychosocial parameters of adolescents and young adults without anxiety and without GJH and the other three groups. A Bonferroni test was used for multiple testing with a significance level of 1.67%. Differences between groups were expressed as mean difference (MD). 

Linear regression was performed to study the impact of GJH and anxiety on physical and psychosocial functioning. Dependent variables consisted of physical factor workload, muscle strength, and psychosocial factors such as fatigue, pain coping, and vigilance. The four subgroups, adolescents and young adults with GJH, adolescents and young adults with anxiety, and adolescents and young adults with GJH and anxiety, were used as independent variables. As potential confounders, age, gender, and BMI were introduced. Data are expressed as regression coefficients, 95% confidence intervals, and r-square.

## 3. Results

All 170 participants from the cohorts 2015 and 2016 were invited to participate. Two students declined to participate, and consequently 168 participants were included (Table 1). GJH was present in 82 (48.8%) participants with higher prevalence in females than males (respectively 56.7% and 35.9%, *p* < 0.01). In total, 16.7% participants suffered from an injury at the time of the screening. Sixty one percent of all participants (68.3% in female and 46.8% in male) showed symptoms of anxiety.

Table 2 shows univariate analysis of psychosocial and physical functioning in adolescents and young adults without GJH and anxiety compared to the scores of adolescents and young adults with GJH or anxiety or both GJH and anxiety. Adolescents and young adults with GJH scored comparable on physical and psychosocial factors to their peers without GJH and without anxiety.

Univariate analysis showed that adolescents and young adults with GJH and anxiety had significant higher scores on fatigue (MD(SE) 11(3.0); *p*-value < 0.01), catastrophizing (MD(SE) 5(1.9); *p*-value 0.01), lower muscle strength (mean difference (MD(SE)−268(57.6); *p*-value < 0.01), and workload (MD(SE)−0.5(0.17); *p*-value < 0.01) than adolescents and young adults without GJH and anxiety. Adolescents and young adults with anxiety alone had significant higher scores on fatigue (MD(SE) 10(3.0); *p*-value < 0.01).

**Figure 1:**
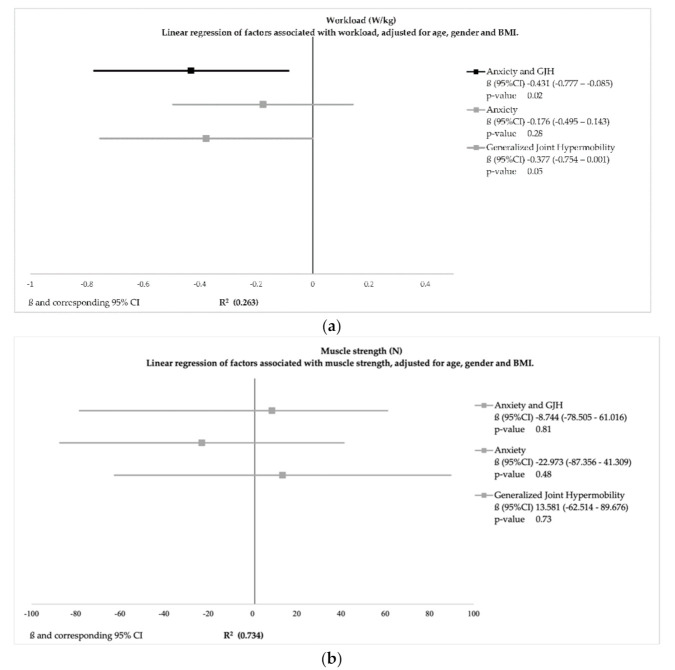

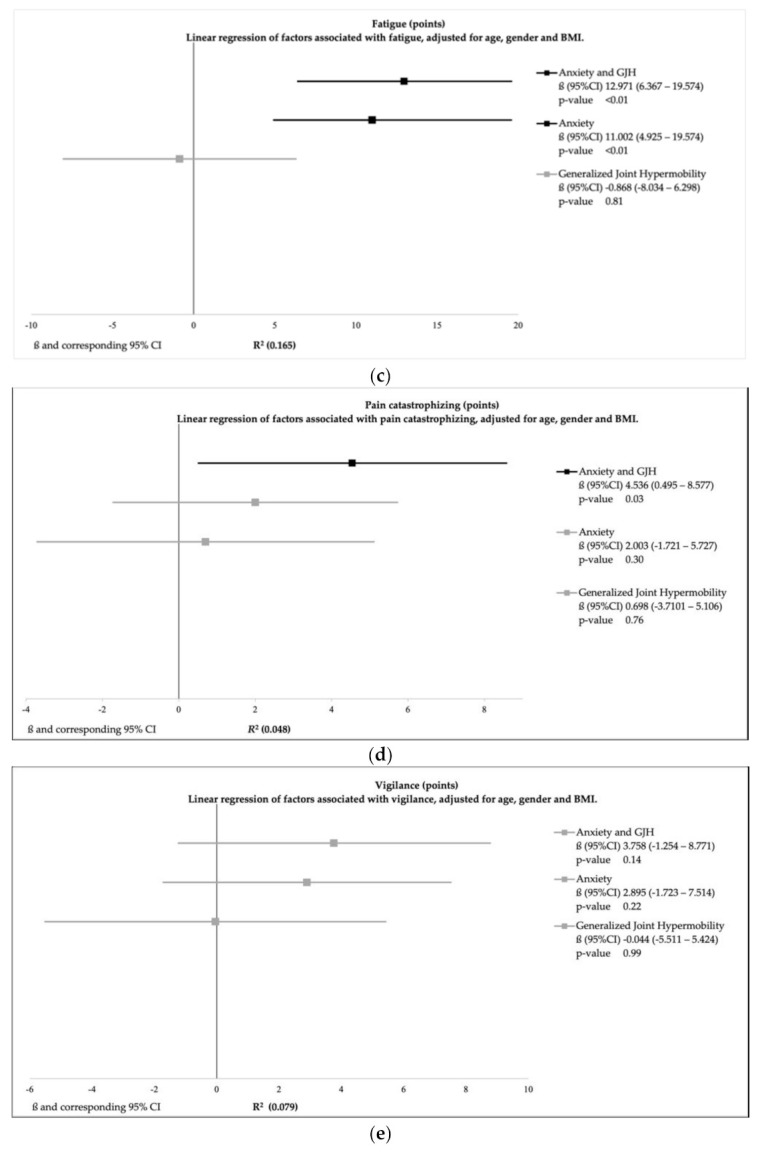
Linear regression models of physical and psychological factors adjusted for age, gender and BMI.

Linear regression models can be found in Figure 1a–e with a R^2^ ranging from 0.05 to 0.73, indicating that a low to relatively high proportion of the variance was explained by the variables.

Linear regression models of psychological factors adjusted for age, gender, and BMI showed that adolescents and young adults with GJH and anxiety had increased scores of fatigue (ß(95%CI) 12.97(6.3–19.5); *p*-value < 0.01) and pain catastrophizing (ß(95%CI) 4.5(0.5–8.6); *p*-value 0.03). Adolescents and young adults with anxiety alone also had increased levels of fatigue (ß(95%CI) 11.0(4.9–19.5); *p*-value < 0.01), while adolescents and young adults with only GJH had no increased risk. Vigilance was associated with age (ß(95%CI) 0.78(0.2–1.1); *p*-value 0.01) and BMI (ß(95%CI) −0.83(−1.6 to −0.1); *p*-value 0.03). Adolescents and young adults with either anxiety or GJH had no increased risk of maladaptive coping or vigilance.

Linear regression analyses regarding physical functioning after adjustment for age, gender, and BMI showed that adolescents and young adults with GJH and anxiety had a significantly decreased workload ((ß(95%CI) −0.43(−0.8–0.08); *p*-value 0.02) (Figure 1). Adolescents and young adults with only GJH had no increased risk. After adjustment, GJH and anxiety, separately or combined, was not associated with muscle strength.

## 4. Discussion

Adolescents and young adults with the combination of GJH and anxiety had decreased workload and experienced more fatigue and pain catastrophizing. GJH alone did not impede adolescents and young adults in their physical and psychosocial functioning.

Current models in the GJH literature [11] describe an association between GJH and anxiety but do not differentiate individuals with both GJH and anxiety from those with only GJH. Our results show that the overall workload was high, representing the physical capacities of our sample as compared to the general population [26]. However, remarkably, within this group, differences existed; a decreased workload was found in adolescents and young adults with both GJH and anxiety compared to their peers without GJH and anxiety. Circumstances in this phase directly before the start of their new education can be perceived as highly demanding, especially by those vulnerable to anxiety related problems. Challenging expectations about their physical performance (potentially especially as perceived important by themselves) can trigger a negative impact of pre-existent psychosocial factors, such as uncertainty, anxiety, or fear. Presence of fear (f.e. fear of failure, fear of injury) and psychological stress in these high performers can induce fear avoidance behavior, which may have influenced maximal performance of individuals with GJH and anxiety. Performance avoidance goals were also observed by Lench et al. in participants with high trait anxiety [27], and a recent review on hypermobile adolescents and young adults proposed that vulnerability to anxiety might cause avoidance behavior as a coping strategy to avoid injury and complaints [9]. In addition, GJH is highly prevalent in the general child population and considered common during growth [28]. Some adolescents develop primarily musculoskeletal complaints, and GJH is associated with functional disability [29]. The adolescents and young adults with GJH and anxiety in our sample did not experience disability; however, they were not able to perform physically equally to reference values.

Furthermore, the presence of increased experience of fatigue, anxiety, and pain catastrophizing in adolescents and young adults with GJH and anxiety compared to adolescents and young adults with anxiety alone who only showed increased experience of fatigue, could suggest that they might already have been struggling to keep up. This suggestion is supported by the literature with different types of anxiety disorders in adolescents showing that somatic symptoms are associated with anxiety severity and impairment in life [30,31].

Within GJH and hEDS/HSD studies, the primary focuses are often on physical symptoms in order to explain physical functioning. Remarkably, we found in our study sample a high percentage of symptoms of anxiety that seems to impact physical functioning even more. The present study has several limitations. First, the external validity of our results: We included a sample of high performers (pre-professional dancers) due to their known high prevalence of GJH. They were selected on a high-performance level of physical and psychosocial functioning but have not started their education. Literature regarding Dutch high performers from the Netherlands Olympic Committee, Netherlands Sports Confederation describe a high incidence of symptoms of anxiety of 57%, comparable to the prevalence in our sample [32].

It is tempting to relate anxiety to the risk of injury these adolescents and young adults might have. In the literature, this well-known concept is referred to as pain related fear and is frequently described in pain populations. The (interpersonal) fear avoidance model is expressing that fear could result in a vicious circle of a psychological trait causing avoidance and eventually could lead to de-conditioning [33]. Our findings also show that participants with anxiety but no hypermobility do have an increased level of fatigue but no pain catastrophizing nor decreased physical functioning. This suggests that the anxiety seems to contribute to a psychological component of fatigue but without restraints for physical capacity and coping in adolescents and young adults without hypermobility. Moreover, our sample is currently starting a professional dance education program, and therefore avoidance or disuse seems not evident within our sample at this specific timepoint based on their choice to participate in an educational program at a dance academy. However, it is possible that participants with GJH and anxiety did show avoidance within our measurements.

Secondly, the cross-sectional design and the specific timing of the measurement does not allow us to draw any conclusions about future dysfunction or potential drop-out. Follow up of these adolescents and young adults seems recommended.

Thirdly, all participants were healthy adolescents and young adults and, so far, successfully completed their audition and are waiting to start their education. Most of the individuals with GJH mentioned within the screening that they were aware of their joint hypermobility, but none of the subjects was diagnosed with an anxiety disorder by a psychologist or psychiatrist. In our sample, the HADS was used to indicate an anxious state in a group of high performers in circumstances that can be perceived as high demanding and thereby stress provoking. These situations are normal to this population; however, the HADS is designed to indicate an anxious state, not to diagnose a psychiatric disorder [34].

For external validation of our results, it is recommended to study the level of functioning in subjects with GJH alone compared to those with GJH and anxiety. To our knowledge, this has not yet been performed in healthy participants nor in participants with a clinical condition.

GJH and anxiety is found to decrease workload, increase levels of fatigue, and enhance pain catastrophizing. However, our results emphasize the importance of longitudinal studies to test whether the combined presence of GJH and anxiety could be used as a predictor for a dysfunctional trajectory or disease progression. Based on our result, we recommend that screening for anxiety is relevant in adolescents and young adults with GJH in high performers as well as in adolescents and young adults with G-HSD and h-EDS, since this might influence tailored interventions.

## 5. Conclusions

Adolescents and young adults with the combination of GJH and anxiety were significantly impaired, showing decreased physical and psychosocial functioning with decreased workload, increased fatigue, and pain catastrophizing. The presence of GJH alone had no negative impact on physical and psychosocial functioning. This study confirms the association between GJH and anxiety. Screening for anxiety is relevant in adolescents and young adults with GJH and might influence tailored interventions.

## Figures and Tables

**Table 1:** Characteristics of all participants (*N* = 168) by gender.

Group Characteristics	All (*N* = 168)						*p* Value
	Male (*N* = 64)			Female (*N* = 104)			
Physical Factors							
Age (mean, SD, range)	20.4	(3.0)	16–29	19.7	(2.9)	15–29	0.12
Weight, kg (mean, SD, range)	67.8	(8.2)	54–91	57.4	(8.4)	39–100	<0.01 *
Height, m (mean, SD, range)	1.78	(0.06)	1.65–1.91	1.66	(0.06)	1.48–1.85	<0.01 *
BMI, kg/m^2^ (mean, SD, range)	21.4	(2.0)	17.8–29.2	20.9	(2.7)	15.8–30.2	0.08
Beighton ≥ 5 (*N*, %)	23		36%	59		57%	<0.01
Wpeak (W/kg), (mean, SD, range)	6.0	(0.7)	4.4–7.5	5.2	(0.76)	2.45–7.24	<0.01 *
Watt max, W, (mean, SD, range)	407	(56)	280–520	298	(57)	106–215	<0.01 *
Strength, Newton, (mean, SD, range)	1843	(174)	1525–2263	1386	(170)	889–1699	<0.01 *
**Psychosocial Factors**							
Anxiety (median, 25th and 75th percentile)	8		1–14	9		0–14	0.06
Symptoms of Anxiety (*N*, %)	31		47%	72		68%	
Depression (median, 25th and 75th percentile)	3		0–14	4		0–10	0.80
Symptoms of Depression (*N*, %)	5		8%	6		6%	0.60
CIS 20 total score (median, 25th and 75th percentile)	50		23–89	52		20–89	0.60
Helplessness (median, 25th and 75th percentile)	5		0–15	4		0–15	0.31
Rumination (median, 25th and 75th percentile)	5		0–18	5		0–15	0.30
Magnification (median, 25th and 75th percentile)	3		0–10	3		0–10	0.24
Vigilance (median, 25th and 75th percentile)	37		7–60	37		12–58	0.78

*N*, number of participants; BMI, body mass index; Wpeak, workload; Watt max, maximum wattage on the steep ramp test; CIS20, Checklist Individual Strength; PCS, Pain Catastrophizing Scale; SD, standard deviation; kg, kilogram; m, meter; %, percentage; W/kg, wattage per kilogram; P50 (25–75); * *p*-value < 0.05.

**Table 2:** Univariate analysis of scores on psychosocial and physical functioning in participants with anxiety and/or GJH and *p* values compared to participants of the No GJH No Anxiety group (Bonferroni correction).

Variables	No GJH No Anxiety (*N* = 38)		GJH (*N* = 27)			Anxiety (*N* = 48)			GJH and Anxiety (*N* = 55)			
					*p* Value			*p* Value			*p* Value	K-W
												*p* Value
Age (mean, SD)	21.3	(3.4)	19.4	(2.1)	0.013	20.4	(2.9)	0.168	19.1	(2.6)	0.001 *	<0.01 *
Females, (*N*, %)	16	42%	16	59%	0.173	28	58%	0.135	44	80%	<0.001 *	<0.01 *
**Physical Factors**												
Weight, kg (mean, SD)	66.6	(11.4)	60.3	(8.9)	0.021	62.9	(7.9)	0.082	56.6	(7.6)	<0.001 *	<0.01 *
BMI, kg/m2 (mean, SD)	22.3	(2.5)	20.8	(2.6)	0.021	21.6	(2.2)	0.190	19.7	(1.9)	<0.001 *	<0.01 *
Strength (mean, SD)	1701	(284)	1583	(250)	0.087	1582	(264)	0.047	1433	(257)	<0.001 *	<0.01 *
Wpeak (Wpeak/kg) (mean, SD)	5.8	(0.82)	5.4	(1.0)	0.098	5.6	(0.8)	0.168	5.3	(0.8)	0.002 *	0.01
**Psychosocial Factors**												
CIS 20 total score (median, 25th and 75th percentile)	46	23–73	46	25–62	0.462	57	21–89	0.001 *	59	20–89	<0.001 *	<0.01 *
Vigilance (median, 25th and 75th percentile)	37	7–59	35	12–60		37	22–58		40	9–59		0.36
PCS (median, 25th and 75th percentile)	11	0–40	12	0–26	0.698	13	0–38	0.266	16	0–31	0.012 *	0.03 *

*N*, number of participants; BMI, body mass index; Wpeak, workload; CIS20, Checklist Individual Strength; PCS, Pain Catastrophizing Scale; SD, standard deviation; kg, kilogram; m, meter; %, percentage; W/kg, wattage per kilogram; P50 (25–75); K–W, Kruskal–Wallis to compare all groups; *p* value display the difference with the reference group; No GJH, no anxiety; * *p*-value < 0.0167.

## Data Availability

The data presented in this study are available on request from the corresponding author. The data are not publicly available due to privacy restrictions.
